# Second-Look Surgery for Colorectal Cancer: Revised Selection Factors and New Treatment Options for Greater Success

**DOI:** 10.1155/2011/915078

**Published:** 2010-12-05

**Authors:** Paul H. Sugarbaker

**Affiliations:** Washington Cancer Institute, 106 Irving Street, NW, Suite 3900, Washington DC,WA 20010, USA

## Abstract

Proper indications for second-look surgery in patients with colorectal cancer have always been a controversial subject. The surgical literature suggests benefit in a reoperation, where a limited extent of cancer is discovered and then resected with negative margins. However, patients are often subjected to a negative exploratory laparotomy or an intervention that is unable to achieve an R-0 resection; in these circumstances, little or no benefit occurs. Unfortunately, an unsuccessful repeat intervention may place the patient in a worse condition, especially if morbidity occurs. This paper seeks to identify the clinical parameters of a primary colorectal cancer and a followup plan that are associated with cancer recurrence that can be definitively addressed by the second look surgery. New surgical technologies, including cytoreductive surgery with peritonectomy and perioperative intraperitoneal chemotherapy with hyperthermia, are suggested for use in this group of patients. This new management strategy used in patients with local-regional recurrence may result in a high proportion of patients converted from a second-look positive patient to a long-term survivor.

## 1. Introduction

Second-look surgery in colorectal cancer patients who develop progression after the surgical removal of the primary malignancy has been extensively explored in the surgical literature [1–5]. Indications for a second intervention in colorectal cancer patients have prompted multiple presentations at international meetings and have always been the subject of much debate. Wangensteen and colleagues organized a planned approach to reoperative surgery in asymptomatic gastrointestinal cancer patients and conscientiously critically evaluated the long-term results [6]. As their clinical experiment evolved, a group of symptomatic second look patients who had clinical evidence of recurrent disease were added to the planned second look in patients who had no signs or symptoms of progressive disease. Minton and colleagues revisited the problem of patient selection for second-look surgery by using an elevation of carcinoembryonic antigen (CEA) obtained at one- to two-month intervals postoperatively to select patients for second-look surgery [7]. 

These paper constitute an important part of the evolution of colorectal cancer surgery. Griffin and colleagues (reviewing the Wangensteen effort) and Minton and colleagues insist, in their summary statements, that symptomatic and asymptomatic second-look surgery in patients with colorectal cancer must be considered as an important option in patient management and constitutes the major rationale for meticulous follow-up management strategies [6–8]. Unfortunately, when these early studies were performed, the repeat surgical intervention occurred without special treatments; their reoperative surgical technology was limited to a more aggressive surgical extirpation of recurrent cancer. Oftentimes, documentation of progressive disease at the time of second-look exploration was not followed by potentially curative surgical extirpation. 

Despite much discussion and multiple publications, the modern revisions of the indications for second-look surgery have never been forthcoming. Wangensteen and colleagues suggested reoperation based on lymph node positivity at the time of primary colon or rectal resection. Minton sought to more effectively select his patients for second look by using a progressively rising CEA blood test. However, patients operated on for symptoms (rather than progressively rising CEA blood tests) had the same benefits at second-look surgery. In both of these clinical experiments, some patients were found to be disease-free (negative second look) and suffered from the morbidity and even an occasional mortality associated with repeat laparotomy. Also, in too many patients, the disease had progressed to an unresectable state. The indications for second look did not select out those patients most likely to benefit from reoperative surgery. 

This paper seeks to identify the clinical parameters and follow-up strategies of a primary colorectal cancer that are associated with a pattern of treatment failure that can be definitively addressed with a second-look surgery. We identify the clinical features of the primary disease process that will allow the cancer surgeon to most knowledgeably select patients for a successful second-look surgery. Also, the use of tumor markers and CT in follow-up are integrated into follow-up regimens. In addition, we identify more effective treatment options for colorectal rectal cancer eradication in the operating room at the time of reoperation. The use of cytoreductive surgery with peritonectomy combined with perioperative hyperthermic chemotherapy are presented as a new and more effective combined management plan to be added to the reoperative surgery. Documentation of improved survival of a subset of patients is the goal of this revised approach for the surgical evolution of second-look surgery in colon and rectal cancer patients.

## 2. A Short History of Second-Look Surgery for Colorectal Cancer

In 1948, Wangensteen at the University of Minnesota initiated a new plan for improved management of intra-abdominal cancer [9]. He reasoned that surgery was the only effective tool by which to cure primary gastrointestinal cancer; therefore, more radical cancer surgery could be used to extirpate isolated sites of disease progression and thereby improve survival. He identified lymph node-positive disease at the time of primary cancer resection as an indication for a systematic plan of reoperation. Every six to eight months, surgical reexploration of the abdomen and pelvis was performed; this reoperation at regular intervals was scheduled until no cancer was found or until the disease was beyond surgical control. Initially, patients were only taken to surgery in an asymptomatic state; however, as the clinical results from the reoperative treatment strategies evolved, patients with symptoms from recurrent disease were included in the series. 

Table 1 summarizes the results of second-look surgery for colon cancer after 20 years of data accumulation. In 36 patients who had a positive second-look, six patients (17%) were “converted” from a second look positive to a disease-free status. In 47 patients who had a symptomatic second look, seven patients (15%) were converted by reoperative surgery to long-term survival. In their review of these data from the second look approach, Griffen and colleagues concluded that “significant patient survival resulted from the second look approach in patients with cancer of the colon. This effort should be continued and extended” [8].

These favorable data regarding patients “converted” from a positive second look to long-term survival were offset, at least in part, by the impact this management plan had on the entire group of patients. Extensive surgical procedures to remove recurrent disease led to an operative mortality of 17% in the patients who had a positive second look. Also, in the patients with the symptomatic second look, there was a 15% operative mortality. In interpreting these formidable mortality statistics, it must be pointed out that this high operative mortality occurred in patients identified as having progressive disease. These patients would presumably have gone on to die of this disease in the near future. However, their lives were cut short as a result of the operative mortality. 

Perhaps most damaging to the concept of a planned second look were the two patients (3.2%) who died postoperatively after a negative exploration. These patients dying with a negative second look may have been long-term survivors in the absence of this aggressive surgical treatment strategy. 

There have been efforts to refine the indications for second-look surgery. In the mid-1970s, a collaborative effort of the Peter Bent Brigham Hospital and the Mallory Gastrointestinal Laboratory searched for clinical relationships between the carcinoembryonic antigen (CEA) and the natural history of surgically treated colorectal cancer. Sugarbaker and colleagues determined that serial carcinoembryonic antigen assays determined at 3 monthly intervals after a colon or rectal cancer resection would detect occult recurrent disease approximately 6 months prior to clinical signs and symptoms [10]. These results led to a clinical study to use CEA as a surveillance test for recurrent colon or rectal cancer after a potentially colon or rectal cancer resection. Steele and colleagues reported on a prospective study of 75 patients who had CEA assays performed at approximately 2-month intervals. Fifteen of 18 tumor recurrences were first diagnosed by increasing CEA values despite no other evidence of progressive disease. In 4 of these 15 patients, complete resection of recurrent cancer with a potential for cure was reported. Long-term follow-up of the total number of patients “converted” from recurrent disease to long-term survival was not available [11]. 

Attiyeh and Sterns collected data on 32 patients who underwent second-look surgery for “a significant CEA elevation” following a curative resection for adenocarcinoma of the large bowel [12]. Not surprisingly, the total number of patients from which these 32 with a rising CEA blood test were selected is not given available. These 32 patients had 37 exploratory procedures with 16 of 37 (43%) potentially curative resections. Again, the number of patients “converted” from recurrent disease to long-term survival was not available from this paper. 

Perhaps the most reliable data regarding the possible benefits of second-look surgery in colorectal cancer patients comes from the prospective evaluation of this strategy reported in 1985 [7]. Minton and colleagues initiated a multi-institutional study. They prospectively collected data on 400 patients operated on at 31 different institutions. His paper emphasizes that the surgeons performing the second-look procedure had advanced training in reoperative surgery. These 400 patients had 43 carcinoembryonic antigen-directed reoperations and 32 symptomatic second-look reoperations. At 5 years following the second look, 22 of 75 patients (29%) remained disease-free at 5 years. Minton and colleagues, as a result of their study, recommended meticulous surveillance of Dukes stage C2 cancer following primary colorectal cancer resection, CEA determinations at 1- to 2-month intervals postoperatively, and reoperation before the serial CEA test exceeded 11 ng/ml. 

As a result of this and other efforts to use postoperative monitoring of CEA in patients at high risk for recurrence of colon and rectal cancer, a standard of practice has evolved in patients surgically treated for colorectal cancer. If a progressive rise in the CEA blood test occurs, patients should be considered for reoperation. Radiologic tests should be performed to show that the elevated CEA determination occurs in the absence of systemic disease or unresectable disease in the abdomen or pelvis. Minton and colleagues should be credited with establishing a strong rationale for meticulous follow-up of colorectal cancer patients and a reasonable likelihood of benefit from second-look surgery.

Goldberg and coworkers pooled data in a prospective follow-up of 1247 patients with resected stage 2 or stage 3 colon cancer [13]. 548 patients had recurrence of colon cancer, and second-look surgery was attempted in 222 patients (41%). In 109 patients (20%), potentially curative surgery resulted. In patients who came to curative intent second-look surgery, recurrent disease was identified by radiologic follow-up in 36 patients, serial CEA tests in 41 patients, and symptoms in 27 patients. The 5-year disease-free survival of these 109 patients was 23%. The surgical mortality was 2%. These authors concluded that postoperative follow-up testing of patients with colon cancer may identify recurrent disease in some and that the second-look surgery can result in a long-term disease-free survival.

## 3. Pathobiology of Colorectal Cancer Recurrence after Surgical Resection

There can be no doubt that the second-look approach initiated by Wangensteen established a new paradigm for a reoperative approach to patients with colorectal cancer. Also, the data on sites of recurrence carefully collected by this group has had a profound effect upon the surgical technology used for primary colorectal cancer resection. Gunderson and Sosin reported the anatomic sites of surgical treatment failure that were identified at the time of reoperation in 74 patients [14]. They report that distant metastases alone was uncommon (8%) but occurred as a component of treatment failure in 50% of the group. Local failure or regional lymph node metastases occurred in 48% of patients as the only site of surgical treatment failure. In 92%, local failure occurred by itself or occurred in combination with distant metastases. Gunderson's data established that local-regional surgical treatment failure was a significant part of the natural history of colorectal cancer.

In a historically important paper that had an influence on surgical technology for colorectal cancer resection, Lofgren et al. examined the pattern of surgical treatment failure in a special group of 47 patients. These patients had a local-regional recurrent disease but were lymph node negative within the primary cancer specimen [15]. From these observations, Lofgren and coworkers reasoned that a mechanism of treatment failure other than lymph node metastases must have caused recurrent disease in these patients. Forty-one of these 47 recurrences were within the operative field and adjacent to or involved the suture line. They reasoned, and this was a novel explanation in 1957, that cancer cells from the primary tumor were left behind and disseminated by the surgeon at the time of removal of the primary lesion. Prior to this publication, colorectal cancer recurrence was thought to be limited to hematogenous and lymphatic dissemination. These new concepts regarding surgical treatment failure led to the promotion of no-touch isolation technique and the total mesorectal excision [16, 17]. 

**Figure 1:**
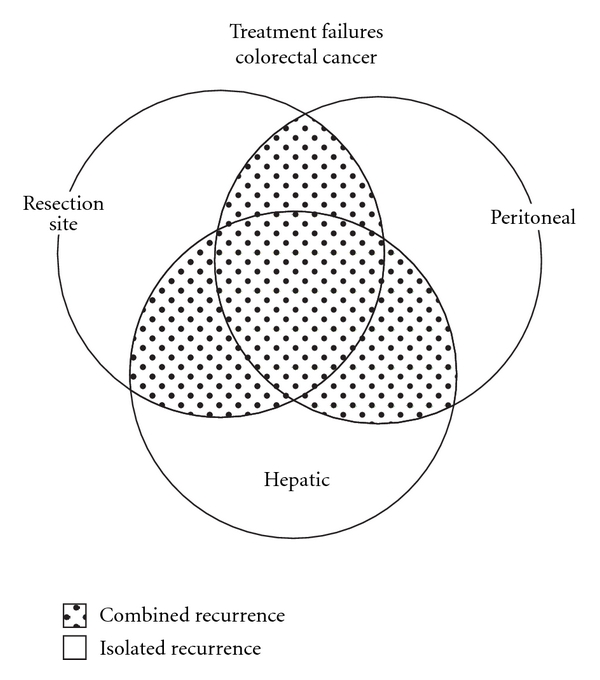
Ring diagram shows that hematogenous metastases and cancer seeding can occur in isolation or in combination with other sites of surgical treatment failure. High-density seeding at the resection site causes a layering of cancer; low-density seeding at a distance results in peritoneal carcinomatosis (reprinted from [18] with permission).

As shown in Figure 1, colorectal cancer recurrence can be attributed to hematogenous dissemination, usually to the liver, intracoelomic dissemination at the resection site as local recurrence, or within the peritoneal cavity as carcinomatosis. Progression of lymph nodal disease causing para-aortic lymph node metastases is an unusual pattern of dissemination. As indicated by the ring diagram, these mechanisms of treatment failure can occur in isolation or in combination [18]. There can be no doubt that as a result of data accumulated at the time of second-look surgery, local recurrence and peritoneal seeding are important anatomic sites of disease progression of surgically treated colon and rectal cancer. 

Sugarbaker and coworkers suggested that peritoneal carcinomatosis and local failure are caused by the same mechanism [19]. Surgical trauma causing dissemination of cancer cells from the primary malignancy is an unfortunate but common occurrence with the cancer resection. Free intracoelomic cancer cells can result from surgical trauma to the primary tumor, blood, or lymph contaminated by cancer cells being spilled into the resection site or free peritoneal cavity or from cancer cells disseminated prior to or at the time of cancer resection from full thickness invasion of the bowel wall. These spilled cancer cells can accumulate at the resection site usually in high density and result in local recurrence. They can accumulate at lower density at distant sites within the peritoneal cavity as peritoneal carcinomatosis. For example, in a rectal cancer, the hollow of the sacrum would be favored by gravity for accumulation of the largest number of cancer cells. Consequently, if the primary rectal cancer is traumatized, a large proportion of patients will suffer a local recurrence. The confines of the pelvis make full exposure difficult so that surgical trauma to the primary cancer is a great danger. The traumatized primary cancer may only lose a few cells, but they would be expected to stick, implant, and then vascularize within the adjacent tissues. By this theory, the resection site of a cancer is at great risk for progressive disease. 

However, not all the cancer cells disseminated from the primary malignancy must implant locally. Some may gain access to the free peritoneal cavity. Cancer cells within intraperitoneal blood clot will later be organized as recurrences within abdominal adhesions. Free cancer cells will move along with peritoneal fluid to distant sites such as the pelvis, beneath the right hemidiaphragm, or in the right and left paracolic sulcus. With small amounts of intraperitoneal fluid, peritoneal seeding proximal to the resection site is expected. With larger amounts of ascites fluid or with mucinous cancer cells, distant spread of carcinomatosis will occur. 

Cancer cells may enter the portal venous system and cause liver metastases. However, cancer cells present within venous blood spilled at the time of primary cancer resection may find their way into the free peritoneal cavity. These free cancer cells may cause resection site recurrence or carcinomatosis.

## 4. New Treatment Options Available for Second-Look Surgery

Second-look surgery has a much greater likelihood of success now than in the past. The knowledgeable use of systemic chemotherapy has reduced the proportion of patients who succumb to progression of systemic micrometastatic disease. The surgical technology for liver resection of colorectal metastases has evolved over the last two decades; liver secondaries are resected if the patient can be made clinically disease-free by liver surgery with minimal morbidity and almost no mortality [20]. Also, surgical technologies for managing local recurrence and peritoneal seeding within the abdomen and pelvis have become available. Visceral resections, peritonectomy procedures, and hyperthermic intraperitoneal chemotherapy offer a treatment option that may be successful in as many as 50% of patients with carcinomatosis [21]. There can be no doubt that the more limited the extent of carcinomatosis, the larger the benefit from definitive treatment.

Extended lymph node dissections so that all possible sites for lymphatic dissemination from colon or rectal cancer are now advocated [22]. Although segmental resections of a colon cancer may have been popular a decade ago, new data strongly supports the surgical removal of all regional lymph nodes that drain the primary cancer. Countless lymphatic channels must be transected in a colon or rectal cancer resection. Cancer cells in lymphatic channels may be lost into the abdominal or pelvic space. A logical conclusion from this hypothesis is that local recurrence is more frequent in patients with colorectal cancers and lymph node metastases [23]. In primary colorectal cancer resection and in reoperative surgery, wide dissection of the colorectal mesentery to the superior mesenteric artery and vein on the right or aorta on the left is indicated.

## 5. New Indications for Second-Look Surgery

The new concept in colorectal cancer resection demands complete clearance of the primary malignancy and total containment of the malignant process during the colon or rectal cancer resection [24]. Unfortunately, there are groups of patients who will fail even the most knowledgeable and skillful efforts to deal definitively with the primary cancer. Also, some patients (approximately 20%) will show peritoneal dissemination of the primary cancer at the time of initial diagnosis [25]. The patients who are at high risk for implantation of cancer seedlings either as local-regional recurrence or peritoneal carcinomatosis can be identified through a careful evaluation of the clinical presentation of the primary cancer and a study of the pathology report. Of course, patients who present with peritoneal dissemination at the time of primary cancer resection will consistently show progression of carcinomatosis. The high-risk patients include mucinous T3 and all T4 cancers, cancers with adjacent organ involvement, cancers with limited peritoneal seeding, cancers with ovarian involvement, cancers with positive peritoneal cytology, and cancers that are ruptured intraoperatively. Also, patients who have a perforation through the primary tumor are known to have a high incidence of local and regional cancer recurrence [25]. Table 2 lists the patients at high risk for progression of local-regional disease or peritoneal carcinomatosis. These patients can be identified as eligible for a planned second look surgery from their clinical presentation and from their pathology reports in the absence of evidence of recurrent cancer.

One requirement of colorectal cancer surgery currently not standard of practice should be added to primary cancer resection in order to thoroughly evaluate patients for this revised second-look approach. A cytology of the peritoneal surface of the primary tumor (optimally a “touch prep”) should occur prior to a colorectal cancer resection. Also, aspiration of fluid for a cytological study of the space beneath the right lobe of the liver and of the pelvis should occur. Finally, a cytological study of the whole abdomen following the completion of the colorectal cancer resection is important. Patients with cytologically positive colon or rectal cancer are at high risk for death from progressive disease [26]. These patients with a positive “touch prep” or positive cytology are identified as high risk for local-regional recurrence and eligible for second-look surgery.

## 6. Implementation of a New Second-Look Treatment Strategy

With a group of colorectal cancer patients identified who are at high risk for local-regional recurrence and with a new and more effective liver resection and peritoneal surface treatment strategies available, a consistent application of second-look surgery should be clinically tested. This comprehensive treatment plan would include three groups of patients: (1) patients at high risk for recurrence by surgical and pathologic findings, (2) patients in the follow-up with symptoms or signs that suggest disease progression, and (3) patients that show a progressive rise in CEA. However, in order to keep the morbidity and mortality with this reoperative surgery at a minimum, ineligibility requirements for this group of patients should be enumerated. Table 3 lists those patients who are not considered reasonable candidates for the revised second-look strategy. Patients with more than four liver metastases or liver metastases that cannot be resected are ineligible. Of course, patients who have a poor performance status or serious medical conditions and are not likely to survive the second-look surgery should not be included. Patients who have had a low rectal cancer with recurrence at the resection site are not good candidates for cytoreductive surgery and hyperthermic intraperitoneal chemotherapy and are poor candidates for reoperation [27]. Of course, patients who have unresectable systemic disease (especially disease in the bone marrow or numerous pulmonary metastases) are not candidates for second-look surgery. The most modern radiologic technologies for detecting systemic disease should be used prior to a reoperative event. This would include CT scan of the chest, abdomen, and pelvis and PET scan.

The final ineligibility requirement listed in Table 3 concerns the interval between primary cancer resection and the second look. In patients at high risk for local-regional recurrence, the intervention should occur within 1 year of the primary cancer resection. This time limitation is an attempt for this clinical pathway to identify patients with disease recurrence that has a limited progression within the abdomen and pelvis. For those patients with peritoneal carcinomatosis, it seeks to identify patients with a peritoneal cancer index of less than 10. There is no doubt that one of the most important requirements for success with cytoreductive surgery and hyperthermic intraperitoneal chemotherapy is a low peritoneal cancer index [28]. 

There is some data regarding a timely second-look surgery in colorectal cancer patients at high risk for local-regional recurrence [29]. Elias and colleagues reported on 29 patients who had no radiologic evidence of recurrence of colon cancer who were taken back to the operating room because of the gross and microscopic findings at the time of primary colorectal cancer resection. The planned second look was within one year of the primary resection. In 55% of patients, persistent adenocarcinoma was documented. In patients with documented disease at second look 9 of 16 patients were disease-free at 27 months median follow-up. These are encouraging preliminary data.

## 7. Algorithm for Revised Second-Look Surgery

Prior to the second-look surgery, the patients would have radiologic tests to rule out systemic disease. CT scan of chest, abdomen, and pelvis is suggested. Also, a PET scan is necessary. A colonoscopy to examine the entire colon looking for second primary tumors and suture line recurrence is a requirement. 

**Figure 2:**
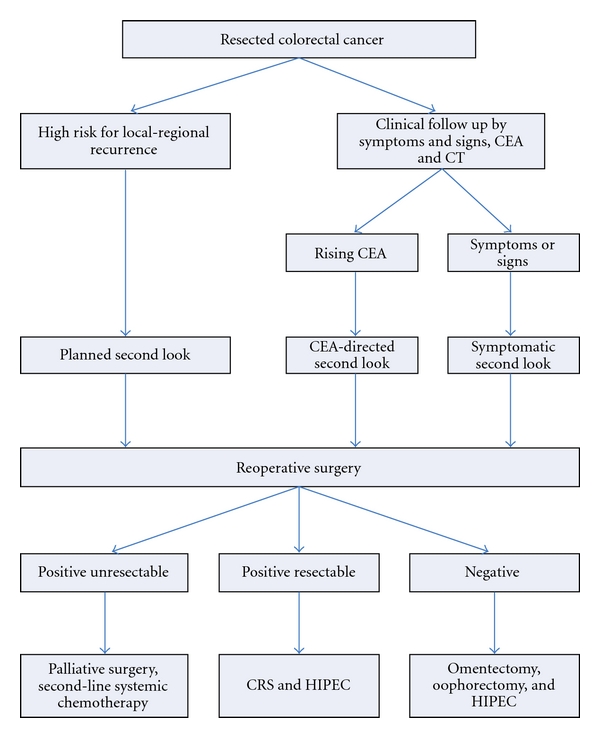
Algorithm for a planned second look in patients at high risk for local-regional cancer recurrence. CEA: carcinoembryonic antigen; CRS: cytoreductive surgery; HIPEC: hyperthermic intraperitoneal chemotherapy.

Figure 2 presents an algorithm for second-look surgery in three groups of patients. There are those patients who have limited carcinomatosis identified at the time of primary colon or rectal cancer resection; also, those patients who, by a study of their clinical symptoms and signs and a review of their pathology report, indicate that they are at high risk for local and regional recurrence [30]. In this group, the maximum time interval between primary colorectal cancer resection and the planned second look should be one year for this plan. Of course, patients with a progressive rise in CEA or symptomatic or radiologic evidence of disease progression will be considered for a second-look surgery. Note in Figure 2 that patients with a negative second look will not have a simple “open and close” laparotomy. These patients are considered at high risk for recurrent disease. In an attempt to protect them from local-regional progression after their full reexploration, they will have a greater and lesser omentectomy, oophorectomy, and a hyperthermic intraperitoneal chemotherapy procedure (HIPEC). It is possible that a limited surgery plus HIPEC will improve the outlook in patients with a negative second look and add little to the expected low morbidity and mortality.

In patients who have a positive second look, there will be cytoreductive surgery which would involve the necessary peritonectomy procedures and visceral resections [31]. Following surgery to remove all visible evidence of disease, hyperthermic intraperitoneal chemotherapy would be performed [32].

## 8. Evaluation of the Revised Second Look

The evaluation of this clinical project must be prospective and thorough. The primary endpoint for the study, which will require approximately a 10-year follow-up, is the percent of patients converted from a positive second look to long-term survival. A secondary endpoint would be the percentage of patients who have a positive second look as compared to those who have a negative second look. A third endpoint is a comprehensive morbidity and mortality assessment of both positive and negative second-look procedures. At the end of data accumulation, a summary of the credits and debits of this approach using the revised second-look surgery will become available (Table 4).

## 9. Timing of Reoperative Surgery, Proactive or Palliative

A large proportion of health care costs occur within the last 6 months of life. For gastrointestinal cancer, recurrence within the abdomen and pelvis allowed to progress to intestinal obstruction, fistula, or abscess brings about a clinical situation which requires extensive and expensive healthcare. Clear guidelines for reoperative colorectal cancer surgery to prevent this devastating clinical situation are not available. Can a reoperative surgical procedure add a reasonable length of good-quality life? Which patients will profit from a surgical intervention and which patients should be told that further surgery is not an option? If a second-or third-look surgical intervention is beneficial when should it be performed? Early, before symptoms occur, or later after patients have lost gastrointestinal function? If the revised guidelines suggested in this paper for second-look surgery are placed into practice, can we prevent local-regional failure in a significant proportion of patients and thereby significantly reduce health care costs? This group of patients at high risk for loss of local control may benefit in terms of quality of life and improved survival by proactive early intervention rather than palliative surgery after symptoms of local-regional disease has occurred.

## 10. Conclusions

A new management plan for patients at high risk for intraabdominal colorectal cancer recurrence is suggested. There are three criteria by which to select patients for a second look: (1) a high risk of local-regional recurrence of cancer, (2) a rising CEA blood test, or (3) symptoms and signs of disease recurrence. Accumulated data shows that early intervention results in a high likelihood of being converted from a second-look positive group to a long-term survivor. New cytoreductive surgical techniques including peritonectomy and HIPEC will be used eradicate abdominal and pelvic recurrence. Also, improved techniques for management of limited liver metastases in combination with peritoneal carcinomatosis are appropriate. From this literature review, the credits and debits for a revised second-look approach to colorectal cancer were formulated. The major credit will be the proportion of patients converted from a positive second look to a long-term survivor. The debits will be morbidity and mortality of the second look for all patients and the cost of the interventions. Important will be morbidity and mortality of those patients who have a negative second look. A projected incidence of positive versus negative second look is 50%. A projected survival of patients with a positive second look converted to a disease-free status is 50% at five years.

## Figures and Tables

**Table 1:** Second-look surgery in patients with cancer of the colon (reprinted from [6] with permission).

	Second-look negative	Second-look positive	Symptomatic look
Number of patients	62	36	47
Operative deaths	2– 3.2%*	6–17%	7–15%
Recurrent cancer^†^	12–19%	24–67%	33–70%
Living and well, last look negative	41	4	4
Living and well, last look positive	—	0	3
Living with residual	1	0	0
Dead from other than cancer	10	2	0
Total converted	—	6–17%	7–15%

*One patient had residual cancer and died at fifth look.

^†^Considered failures in negative-look group.

**Table 2:** Patients with primary colorectal cancer identified to be at high risk for local-regional recurrence and/or peritoneal carcinomatosis.

	(1) Visible evidence of peritoneal carcinomatosis.
	(2) Ovarian cysts showing adenocarcinoma suggested to be of gastrointestinal origin.
	(3) Positive cytology either before or after cancer resection.
	(4) Adjacent organ involvement or cancer-induced fistula.
	(5) Obstructed cancer.
	(6) Perforated cancer.
	(7) T3 mucinous cancer.
	(8) T4 cancer or a positive “touch prep” of the primary cancer.
	(9) Cancer mass ruptured with the resection.
	(10) Positive lateral margins of excision.

**Table 3:** Ineligibility requirements in patients considered for second-look surgery.

*Major:*
Liver metastases >4
Performance status >2
Serious medical condition
Renal failure with creatinine >3
Cardiac failure with ejection fraction <50%
Malnutrition
Radiologic study showing systemic disease

*Minor**: ***
Obesity
Low rectal cancer
Intestinal obstruction from progressive cancer or a long interval
between cancer recurrence and second look

**Table 4:** Endpoints of revised guidelines for second-look surgery.

Credits	Debits
(1) Percentage of patients “converted” from disease recurrence to five-year survival.	(1) Percentage of patients with a negative second look.
(2) Median time to recurrence and median survival of patients with a positive second look who are resected.	(2) Morbidity and mortality of patients with a negative second look.
(3) Median time to recurrence and median survival of patients with a positive second look who are unresectable.	(3) Morbidity and mortality of patients with a positive second look.
	(4) Cost of reoperative surgery.
	(5) Cost of followup program.
